# Racism as a Social Determinant of Mental Health in Higher Education: Sector-Level Perspectives From South Africa and Australia

**DOI:** 10.3389/ijph.2024.1607502

**Published:** 2024-11-22

**Authors:** Xuan Luu, Veena Abraham

**Affiliations:** ^1^ School of Medicine and Public Health, College of Health, Medicine and Wellbeing, The University of Newcastle, Callaghan, NSW, Australia; ^2^ Department of Pharmaceutical Sciences, School of Pharmacy, Sefako Makgatho Health Sciences University, Pretoria, South Africa

**Keywords:** racial disparities, mental health, higher education, social determinants, institutional trust

**The IJPH series “Young Researcher Editorial” is a training project of the Swiss School of Public Health.**

High levels of mental ill-health occur in universities and other institutions of higher education. Considerable numbers of students report anxiety (29%) and stress (23%) [1], and nearly two-thirds of staff report either possible or probable depression [2]. While many universities worldwide are making efforts to improve student and staff mental health [3], their approaches focus largely on changing lifestyle factors at the individual level [4]. However, mental health is also influenced by large-scale social and structural factors — such as racism [5]. In this editorial, we argue that universities and higher education sectors should address racism as a social determinant of mental health. We support our argument with sector-level perspectives from two distinct regions: South Africa and Australia.

In South Africa, systemic racism is a lingering legacy of apartheid. While Historically White institutions of higher education are well-funded to implement policies and programmes to support mental health, there are continuing financial, resource, and infrastructure constraints that impact Historically Black institutions [6]. Moreover, Black academic leaders who work in white institutions face structural racism that taxes their resources and harms their mental health; the “Black academic tax” imposes upon Black staff the duties of driving change, acting as role models, supporting Black students and colleagues, and combatting covert racism that pervades the structures of their institutions. For example, an independent inquiry identified deep-seated institutional racism as a major stressor following the 27 July 2018 suicide of Professor Bongani Mayosi, who was employed at a well-established Historically White institution [7].

In Australia, a recent review of the nation’s higher education system identified structural racialised barriers and interpersonal racism as institutional failures that erode health and wellbeing — particularly among First Nations (i.e., Indigenous) students and staff, as well as students and staff from other racially marginalised and minoritised groups [8]. While Australian universities have publicly stated commitments to addressing racism [9], little research has examined people’s experiences of racism on campus and there is a need for greater institutional accountability [10]. Some small steps are now occurring in response. For instance, the Australian Human Rights Commission has received $2.5 million in national government funding to lead an independent research study on the prevalence and impact of racism in universities — intended as a foundational source of evidence to guide institutional action [11].

Based on sector-level perspectives across both South Africa and Australia, comprehensive action is needed to address the pervasive and persistent problem of racism in universities. Tackling racism as a social determinant of mental health in higher education requires institutional change at both the programme and policy levels; while programme-level intervention can address the individual and relational manifestations of racism, policy-level measures are also needed to reduce the systemic barriers and structural inequities that perpetuate racism in institutional contexts [12]. Some priority areas for institutional action have been highlighted in existing literature — including governance and leadership (e.g., making anti-racism a mandatory governance agenda item), operations and processes (e.g., improving policies and processes for reporting racism in university settings), and strategic planning and accountability (e.g., aligning strategic planning and accountability measures with anti-racist principles) [13]. These priorities serve as a key starting point for bringing about change.

Universities need to address the social determinants of student and staff mental health — including racism. Reflecting on insights from South Africa and Australia, the historic and ongoing impacts of systemic racism are clear across both regions. This includes corrosion of the very wellbeing of students and staff who are affected. We call on scholars, practitioners, and decision-makers to understand how racism manifests in their institutions, advocate for programme- and policy-level change, and share evidence of how universities are taking clear, accountable steps to change the conditions that give rise to racism in higher education — all critical to improving the mental health of students and staff.

## Data Availability

The original contributions presented in the study are included in the article/supplementary material, further inquiries can be directed to the corresponding authors.
